# Traveling Imposters

**Published:** 1871-10

**Authors:** 


### TRAVELING IMPOSTERS.

Mr. Jeffrey Smith, of Woodhull, Steuben Co., N. Y., called, recently, and informed us that he had purchased a pair of spectacles of a chap who called himself Dr. MARKS, and who represented himself as an agent of the Elmira Eye and Ear Institute, saying that he had been in our employ for the past ten years. He is an Englishman, about forty years old, and travels from house to house, by means of a horse and buggy. He deals in the poorest sort of spectacles, which he represents to be the finest of "pebble," and disposes of them at prices ranging from five to fifteen dollars.

The pair Mr. Smith purchased for fifteen dollars, we can readily duplicate for fifty cents. He is doing a fine business, having sold several "high priced" glasses in Mr. Smith's immediate neighborhood. Mr. Smith called for the purpose of exchanging his glasses, as he was assured by the Agent (?) that we would furnish him another pair should he so desire.

We have before been called upon to disclaim any knowledge of these traveling imposters, and have repeatedly informed our readers that we do not deal in spectacles, neither do we travel nor send out agents to represent our Institution, in any particular. Any person who is silly enough to patronize a traveling doctor, or spectacle vendor, richly *deserves* to be swindled. No respectable physician will ever be found traveling, if he is what he represents himself to be, upon his flaming posters, and through the public prints, he can find sufficient to do at home. Good physicians will always find plenty of employment without having to resort to traveling from place to place in search of patients; and as for traveling spectacle vendors, they are too ridiculous to be thought of by any intelligent person. A pair of glasses should *never* be worn without first consulting an oculist, and having him decide whether your eyes *require* glasses, and if so, what sort. He prescribes the kind to be worn, and you then purchase them of some *reliable* optician who will supply the needed article, charging therefor a reasonable price. Thousands of eyes are ruined by wearing glasses that are recommended by opticians who have not, nor are they expected to have, any knowledge whatever of the eye or its defects. We are often called upon to examine eyes and to inform the patient *why* glasses do not enable them to see, when such an examination reveals the fact that no glasses are necessary, but, contrariwise, are doing an absolute harm.

Many people suppose that the moment the eye fails to perform a normal amount of vision, that something *must* be wrong with its refractive power, and that therefore spectacles is the remedy They then consult an optician instead of an oculist, and buy a pair of glasses that magnify print sufficiently to enable them to read, and are then stupid enough to suppose that they are doing the proper thing.

If people will ever learn not to patronize traveling doctors, perambulating spectacle venders, or to wear glasses of any sort, without the advice of an oculist, they will have disposed of three great evils that are cursing every community.

